# Applications of carbon dots and its modified carbon dots in bone defect repair

**DOI:** 10.1186/s13036-022-00311-x

**Published:** 2022-11-22

**Authors:** Longchuan Zhu, Weijian Kong, Jijun Ma, Renfeng Zhang, Cheng Qin, Hao Liu, Su Pan

**Affiliations:** 1 Department of Orthopedic Surgery, Second Hospital Jilin University, Ziqiang St 218, 130041 Changchun, People’s Republic of China; 2 Department of Orthopedic Surgery, Baicheng Hospital Traditional Chinese Medicine, Jilin, People’s Republic of China

**Keywords:** Bone defect repair, Carbon dots, Antibacterial, Anti-infective, Good biocompatibility, Alkaline phosphatase, Gene regulation

## Abstract

Bone defect repair is a continual and complicated process driven by a variety of variables. Because of its bright multicolor luminescence, superior biocompatibility, water dispersibility, and simplicity of synthesis from diverse carbon sources, carbon dots (CDs) have received a lot of interest. It has a broad variety of potential biological uses, including bone defect repair, spinal cord injury, and wound healing. Materials including CDs as the matrix or major component have shown considerable benefits in enabling bone defect healing in recent years. By altering the carbon dots or mixing them with other wound healing-promoting agents or materials, the repair effect may be boosted even further. The report also shows and discusses the use of CDs to heal bone abnormalities. The study first presents the fundamental features of CDs in bone defect healing, then provides CDs manufacturing techniques that should be employed in bone defect repair, and lastly examines their development in the area of bioengineering, particularly in bone defect repair. In this work, we look at how carbon dots and their alteration products may help with bone defect healing by being antibacterial, anti-infective, osteogenic differentiation-promoting, and gene-regulating.

## Introduction

In vertebrates, bones are hard organs that exercise, support, and defend the body while also creating red and white blood cells and storing minerals. Mineralized bone tissue, which has a strong honeycomb-like three-dimensional structure within, is one of the components of bone. The skeleton of the human body supports the body and is an essential component of the human motion system. However, the integrity of the bone is readily eroded by many unfavorable events, resulting in abnormalities and the loss of normal structure and function. The capacity of bone regeneration allows a portion of a bone defect to regenerate over time, but the repair process is dynamic and ongoing, and it is readily influenced by a range of external variables. As a result, it is critical for bone damage repair to conduct appropriate intervention and protective measures to offer a proper environment and circumstances for bone defect repair.

Significant fractures and large bone deformities are a difficult issue for orthopedic surgeons to cure. People are trying to load growth factors or other compounds with anti-inflammatory, antimicrobial, and excellent biological activity into scaffold biomaterials using tissue engineering / regenerative medicine to stimulate the healing of bone deformities in today's study. As a result, bone abnormalities were treated using hydrogel [1–3], fiber spinning [4–6], and carbon-based materials [7–9].

Carbon dots (CDs), also known as carbon nanodots (Cnds), are a two-dimensional carbon nanomaterial with a diameter of roughly 10 nanometers [10–12]. Because of its unique physical and optical qualities, CDs are frequently employed in bioengineering-related fields [13–15]. Their discovery may be traced back to research published in 2004 on the composition of fluorescent nanoparticles made from single-walled carbon nanotubes. Since then, CDs have been studied extensively in a variety of domains, including biosensors, biological imaging, drug administration, and optoelectronics.

The fast growth of nanotechnology in recent decades has brought fresh innovations to the area of biomedicine. A range of innovative therapies have emerged in response to conventional medicine's biological problems. Quantum dots have been extensively employed in photocatalysis [16–18], light-emitting diodes [19–21], and ion detection [22–25],notably in biomedicine, thanks to the development of diverse precursors [26–30]. Although the use of carbon dots as a defect repair material may protect the wound to some degree, the carbon dots themselves are defective owing to an insufficient manufacturing technique and a lack of more extensive biological features. As a result, we must change the carbon dots using various preparation procedures to give them new qualities that will aid in the repair of bone defects and promote bone healing. As a result, this study examines the current state of CDs research in bone defect repair, as well as the synthesis and use of CDs in the area of bone defect repair, as well as the various features of CDs in bone defect repair.

This paper carefully covers the characteristics, synthesis techniques, yield and size of carbon dots, as well as the extensive use of carbon dots and their modified products in the biomedical sector, based on a significant number of experimental research. At the same time, this work sums up the use of carbon dots and their modified products in bone defect healing and discusses the features of carbon dots and their modified products in terms of synthesis process and repair mechanism. There are no other papers that provide such a thorough examination of the use of carbon dots in bone defect healing.

## The process and mechanism of bone defect repair

Only when the force surpasses the bone strength, or when the damage gradually accumulates under a cyclic load (far lower than the bone strength), does bone dynamic balance successfully avoid fracture [31–33]. Bone regeneration and repair, unlike other tissues, may be completely returned to pre-injury composition, structure, and function, according to existing research [34]. The degree of tissue loss is one of the elements that drive bone regeneration and may also be used to define bone restoration. As a result, there are two forms of bone repair: primary and secondary.

When the fracture gap is smaller than 0.1mm and the fracture site is securely secured, primary (direct) bone healing occurs. There is no cartilage or connective tissue creation in this phase, and the bone gap is immediately filled by continuous ossification and subsequent Havers remodeling [35], callus development is also susceptible to a number of limitations [36]. It should be mentioned, however, that the idea of continuous bone growth is debatable since no histology evidence or clinical instances exist [33].

Primary healing is only possible for most fractures with solid internal fixation. Inhibit the development of periosteal callus, encourage the creation of primary reactive callus, enable bone marrow circulation and osteogenic tissue passing through the fracture end, and assure bone marrow healing with firm internal fixation. stimulate bone cortical repair or fracture connection, and then proceed to the fracture's secondary healing stage.

**Fig. 1:**
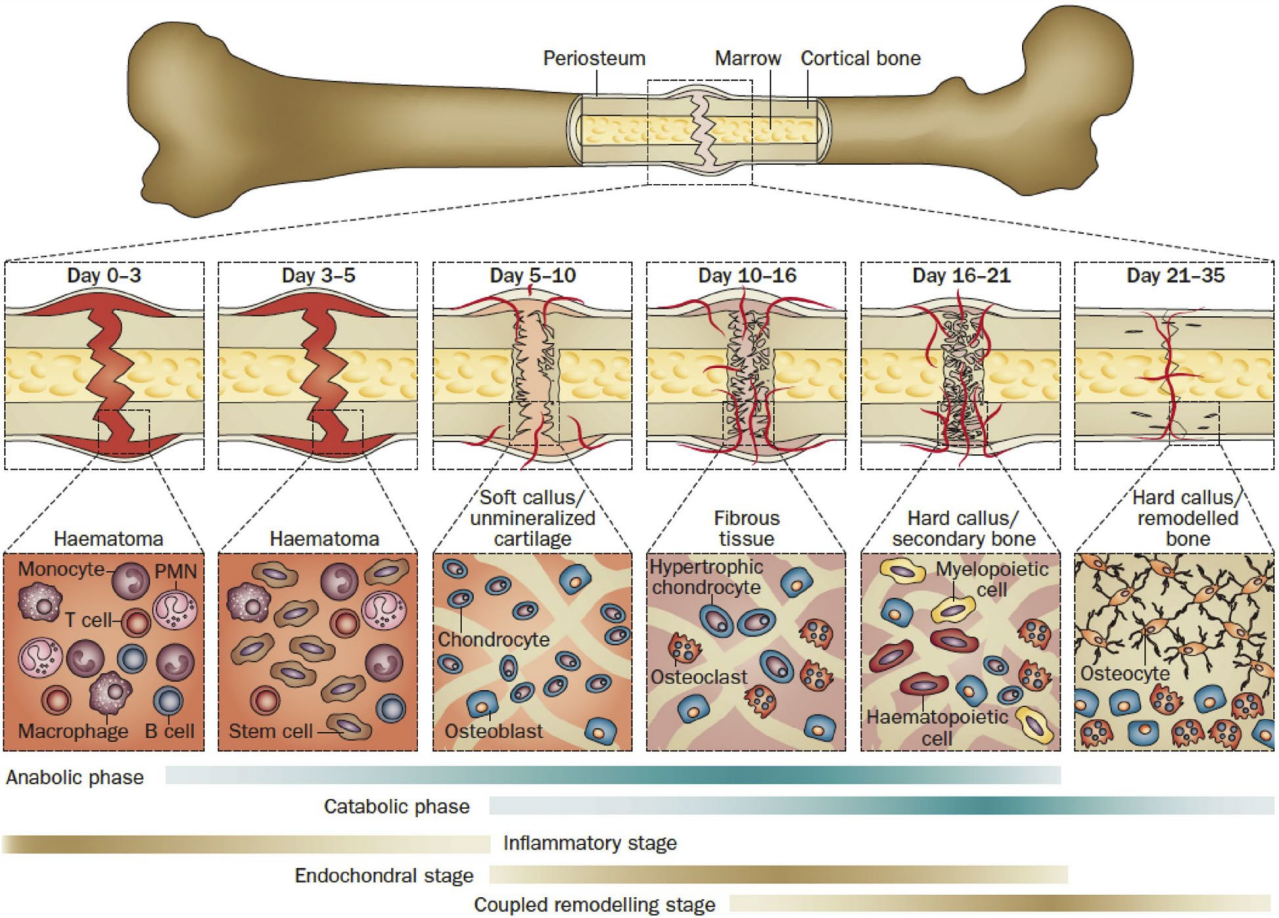
Typical fracture healing process, biological processes, and distinct phases of cell activity. The major metabolic stage of fracture healing (blue strip) coincides with the biological stage (brown strip). In mice, the healing period is equal to that of a closed femoral fracture treated with an intramedullary rod. Abbreviations: BMP, Bone Morphogenetic protein; BMPR, Bone Morphogenetic protein receptor; DKK1,Dickkopf related protein 1; low density Lipoprotein receptor related protein; MSCs, Mesenchymal Stem cells; PMN; parathyroid hormone; parathyroid hormone related protein; RANKL, Nuclear Factor κ B Ligand receptor Activator [34]. (Copyright © 2014, Nature Publishing Group, a division of Macmillan Publishers Limited. All Rights Reserved.)

The most frequent kind of bone healing is secondary (indirect) bone healing, which occurs when the fracture edge is less than twice the diameter of the wounded bone. Coagulation, inflammation, fibrocartilage callus development, intramembranous and endochondral ossification, bone remodeling, and other processes are all involved in secondary fracture healing. Anabolic metabolism in fracture is first stimulated by increasing bone volume, which is accomplished by attracting stem cells to differentiate and preventing chondrocyte death. Anabolic activity, especially catabolic activity, lasts a long time [33]. Figure 1 depicts the timing of fracture healing and cellular changes at various phases.

Large segmental bone defect, also known as crucial bone defect, is a kind of bone healing disorder that may be produced by high energy trauma, illness, developmental deformity, revision surgery, tumor excision, or osteomyelitis [37, 38]. Extensive bone loss in this defect has been found to have a direct impact on revascularization and tissue differentiation, resulting in spontaneous fractures that progress to nonunion without surgery.

Biomaterial scaffolds, cells with osteogenic potential, and growth factors are the three primary components of current therapeutic techniques for bone defect healing. A favorable milieu for bone regeneration is provided by the biomaterial scaffold's nano/micron structure [39]. Nanotopography may directly influence osteoblast lineage cell activity, encourage osteoblast development, and create an advantageous bone immune microenvironment [40–42]. The growth, proliferation, differentiation, mineralization, and final creation of bone may all occur on a basis provided by this microstructural pattern in osteoblast lineage cells.

The use of nano/micron materials in medical therapies has grown significantly in recent years. According to the various spatial scales of biomaterial structures, nano/micron materials, which comprise particles, composites, and surfaces, may be divided into three categories: nanoscale (≤100 nm), submicron (100 nm-1μm), and micron (≥1 m). It has been shown that adding hydrogels containing 10% nanoparticles may improve the mechanical characteristics of composite biomaterials and aid in the development of new bone in animals. In order to stimulate the creation of bone, multilayer cell sheets with various patterns were created by magnetically labeling stem cells with Fe_3_O_4_ nanoparticles enclosed in graphene oxide. Under thermodynamic control, intrafibrillar mineralized collagen with bone-like hierarchical nanostructures (HIMC) was created [43]. The aforementioned research shows how the use of nano/micron materials may provide a favorable milieu for cellular multidirectional differentiation and the production of new bone. Additionally, their high porosity, interconnecting pores, and bone-like nanostructure enable osteoblast migration and vascular development.

Organic and inorganic elements are combined at the submicron scale to generate interwoven nanostructured mineralized collagen fibers in bone tissue, which is a functionally and physically hierarchical system [44]. The millimeter- to nanoscale-scale hierarchical structure of bone includes nanophases, mineralized fibers, and fiber bundles (collagen molecules and mineral particles). As a result, the biomaterials to which we apply must possess complicated multiscale features and the capacity to affect cellular activity both at the molecular and cellular levels.

Cells and the nanostructures of the extracellular matrix interact in complicated ways at the nanoscale, and as a consequence, the nanotopography at the interface may regulate how cells behave [45, 46]. The present study has shown the importance of nanotopography in bone marrow MSC populations and bone differentiation. In this study, nanoparticles having a diameter of 120 nm were created using square arrays (SQ), shifted square arrays (DSQ 50) with points off square 50 nm, DSQ 20 (20 nm off true center), and random insertion [47]. MSCs in the planar control SQ group displayed fibroblast-like form after 21 days in culture, but did not express osteocalcin (OCN) or bone bridge protein (OPN) immunocytochemically. Positive OPN and negative OCN expression accompanied the osteoblastic morphology of DSQ20 cells. OPN and OCN were expressed positively in the DSQ50 group, and MSCs congregated fast to form discrete areas and mineralized nodules. Furthermore, MSCs cultured under random implantation conditions revealed osteoblastic morphology but negative OPN/OCN expression. The aforementioned experiments show how the presence of nanoparticles alters the nanotopography of a material's surface, which in turn causes noticeable variations in cellular response.

In conclusion, it can be concluded that the material utilized to repair bone defects should be based on a nanotopological simulation of natural bone, namely a hierarchical nanostructure created by the interlocking assembly of collagen and nano-hydroxyapatite [43, 48]. A highly branching, "osteoblast-like" morphology with long filamentous pedicles and thick stress fiber production has been shown for cells growing on hierarchical nanostructures [49]. Additionally, the hierarchical nanostructure group had considerably greater expression levels of the transcription factors Runt-related transcription factor 2 (Runx2) and vascular endothelial growth factor [50].

Injectable hydrogels were combined with protein-based nanofiber particles to create a brand-new kind of hydrogel, which helped Hou et al. enhance their biomaterial [51]. Due to the presence of nanoparticles, the hierarchically constructed hydrogel displayed a nanofibrous structure resembling an extracellular matrix. Compared to smooth surfaces, the novel hydrogel offers superior cell adhesion because of its larger specific surface area and ability to bind extracellular matrix proteins including fibronectin and glass-linked proteins. Thus, it can be shown that the potential of nanomaterials for bone defect healing is demonstrated by the application of nanoscale and nanotopographic characteristics to influence the activity of mesenchymal stem cells [52].

The introduction of nanoparticles guides osteogenesis by topographically modifying the local immunological milieu, in addition to creating structures akin to bone tissue. Nanostructures have the potential to alter the cell shape, proliferation, adhesion, and phenotype of macrophages, the key immune cells that mediate biomaterial-related responses. The surface form of nano-needle-shaped calcium-deficient hydroxyapatite is critical in promoting macrophage production of pro-inflammatory cytokines and so directing the osteogenesis process. Furthermore, they demonstrated that specific physicochemical properties of nanostructured biomaterial scaffolds might activate macrophage immune responses [50]. They discovered that bionic graded nano-interfaces may induce M2 macrophage polarization and IL-4 release, promoting stem cell osteogenesis and endogenous bone repair. The use of nanoparticles during bone regeneration may directly affect the activity of osteoblast lineage cells, improve osteogenic differentiation, and produce a favorable bone immune environment, which is extremely promising for bone defect repair.

## Basic properties of carbon dots in bone defect repair

**Fig. 2:**
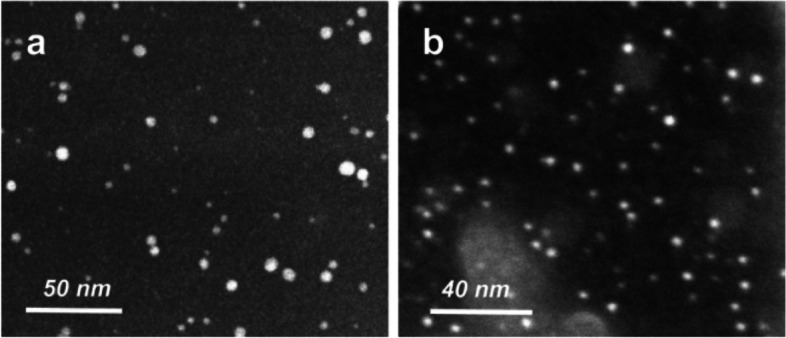
Typical STEM images of (**A**) PEG1500N and (**B**) PPEI-EI surface passivated carbon dots [10]. (Copyright © 2006, American Chemical Society)

CDs are carbon nanostructures with zero dimensions and a size of about 10 nm (Fig. 2). CDs may have sp2, sp3, or sp3 hybridized carbon atoms and can be spherical, crystalline, or amorphous. Broadband absorption and size-dependent photoemission at 260–320 nm, strong photoluminescence quantum yields, a tunable surface with minimal toxicity, and appropriate electron transport properties are all characteristics of CDs. It is a suitable option for biological applications because of these qualities [7].

Recent CD-based studies in bone tissue engineering have become relevant [53]. CDs-based bioscaffolds are thought to be useful materials for bone regeneration and repair. Gogoi et al. created CDs-peptide composites implanted in tannic acid and polyurethane matrix for in vivo bone regeneration. The experimental group supplemented with CDs had the greatest levels of biological activity in terms of osteoblast adhesion, osteogenic differentiation, and cell proliferation [54]. These CDs-based nanocomposites demonstrated better mechanical properties and osteogenic activity in studies using MG 63 osteoblasts. The data suggest that the extraordinary mechanical strength of the scaffolds is attributable to the homogenous distribution of CDs in hydroxyapatite and the cross-linking of CDs and polyurethane.

Several studies have also shown that adequate MSC proliferation is required for the repair of injured or insufficient organs, such as bone tissue. One of the key difficulties in the field of bone tissue engineering is the effective scaffolding of body mineralization. And the right-sized and-shaped CDs may be able to exactly resolve this problem. Shao et al. investigated the influence of CDs on osteogenic differentiation. The data revealed that CDs may significantly increase the mineralization process while also efficiently generating osteogenic differentiation of rBMSCs. Furthermore, CDs have the properties of being biocompatible, non-toxic, and encouraging osteogenic gene expression, making them appropriate as materials for bone defect repair [55].

In conclusion, the unique structure and capabilities of CDs enable innovative medicinal and biological applications. CDs might be one of the best biological applications for bone tissue engineering right now. The low toxicity and ease of manufacture of CDs in bone tissue engineering scaffolds are the most prominent features. Scaffolds for bone regeneration have exceptional mechanical properties due to efficient cellular interaction and CDs cross-linker synthesis. Furthermore, the homogeneous and regular distribution of CDs in the matrix influences cell bioactivity. CDs are unique in comparison to other types of nanostructures in possessing the aforementioned capacity.

## Preparation method of carbon dots in bone defect repair

**Fig. 3:**
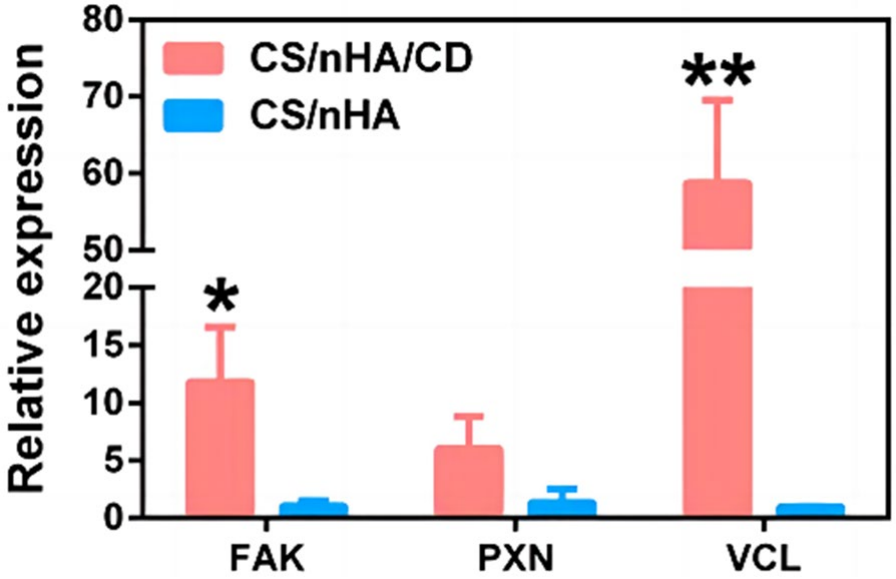
Relative expression levels of cell adhesion-related genes in scaffolds. The expression of the adhesion patch pathway genes FAK and VCL was significantly enhanced in the CS/nHA/CD scaffold. the expression of PXN was also higher in the CS/nHA/CD scaffold [56]

CDs preparation may be separated into two approaches based on the direction of increase of the size of the realized materials: "Top-down" and "Bottom-up" (Fig. 3). These two tactics will be addressed in detail in the following sections. The synthesis of CDs used to repair bone abnormalities will be explored in the parts that follow from both a Top-down and Bottom-up perspective. Table 1 details various synthesis processes, including size, and physicochemical attributes.

**Table 1:** Carbon dots and their manufacturing techniques for the healing of bone defects

Preparation method	Carbon dots type	Surface added functional groups	Size	Properties	Reference
Hydrothermal treatment	HAP-CDs	Hydroxyapatite	20-30nm	Strong cell adhesion and alkaline phosphatase activity	[54]
-	CG- CDs	Collagen-genipin	6-10nm	Promote differentiation of BMSCs to chondrocytes and promote cartilage regeneration	[57]
Hydrothermal cum co-precipitation	HAP-NCDs	Hydroxyapatite	85-200nm	Increased expression of osteogenic-related transcription factor 2, alkaline phosphatase, and osteocalcin Increased bone density	[58]
Microwave-assisted pyrolysis	MiR-CDs	MiR-2861, Ascorbic acid, PEI	~2.5nm	Promotes osteogenic differentiation	[59]
Hydrothermal treatment	Zn-CDs	Zn^2+^	5.25nm	Induce osteoblast differentiation	[60]
Hydrothermal treatment	Zn-CDs	Zn^2+^	1.7-2.5nm	Promotes bone regeneration Has good osteogenic activity	[61]
-	SMCC-CDs	Sulfosuccinimidyl-4-(N-maleimidomethyl) cyclohexane-1-carboxylate (sulfo-SMCC)	13-22nm	Promotes differentiation of bMSCs to chondrocytes Promotes cartilage regeneration in vivo	[62]
Hydrothermal treatment	Fe-CDs	Super-paramagnetic iron oxide nanoparticles	40-60nm	Promotes osteochondral differentiation	[63]
Microwave-assisted	Ascorbic acid- CDs	Ascorbic acid	2-3nm	Activate the PERK-eIF2α-ATF4 signaling pathway Increased BSP and OCN expression Promote pre-osteoblast differentiation and bone regeneration	[64]
Hydrothermal treatment	M-CDs	Metformin	3.76nm	Promotes bone marrow mesenchymal stem cells (BMSCs), alkaline phosphatase (ALP) activity, calcium deposition nodule formation, and expression of osteogenic genes and proteins	[65]
Microwave	CS-NHA-CDs	Chitosan, Nano-hydroxyapatite	5nm	Enhanced adhesion and osteogenic differentiation of rBMSCs, good antibacterial effect Promoted the formation of vascularized bone tissue	[56]
Hydrothermal treatment	SPIC-CDs	SVVYGLR, PRGDSGYRGDS, IPP, CGGKVGKACCVPTKLSPISVLYK	2.2-4.8nm	Stimulates osteoblast adhesion, proliferation and differentiation, and induces angiogenesis	[53]
Hydrothermal treatment	Citric acid-CDs	Citric acid	-	Upregulate the expression of osteoblast gene markers ALP, RUNX2, OCN and BSP to promote matrix mineralization and thus promote osteogenic differentiation of rBMSCs	[55]
Hydrothermal treatment	PCL-CP-CDs	PCL, captopril	~10nm	Enhanced activity, adhesion, alkaline phosphatase activity and mineralization	[66]
Hydrothermal treatment	PCL/PVA-TCP3-CDs	PCL, PVA, calcium phosphate	5-7nm	Significantly increased alkaline phosphatase activity and cell proliferation rate	[67]
Microwave-assisted hydrothermal treatment	WS2 HJS-CDs	Heterojunction, WS2 nanosheets	9.3-11.9nm	Significantly promotes osteogenic differentiation and significantly upregulates the expression of bone-related genes	[68]
Hydrothermal treatment	OH-CDs	Hydroxyl	4nm	Scavenges free radical groups and promotes cell proliferation	[69]
Hydrothermal treatment	AA-CDs	Adenosine, aspirin	2-5nm	Induced differentiation of hBMSCs toward osteogenesis	[70]
Hydrothermal treatment	PLA-CDs	PLA	3-7nm	Promotes cell proliferation, increased bone mineralization, and increased osteogenic differentiation	[71]
Microwave-assisted	p-CDs	Positive charge	3.6-5.8nm	Enhance antibacterial effect	[72]
Microwave-assisted	n-CDs	Negative charge	1.7-4.1nm	Promote osteogenic differentiation	[72]
Hydrothermal treatment	CDs	-	~10nm	High affinity with bone	[73]
Hydrothermal treatment	Mg-CDs	Mg^2+^	Up to 39.8nm with increasing temperature	Increase alkaline phosphatase (ALP) activity Upregulate the expression of osteogenic-related genes: Runx2, OSX, Col1a1, OCN	[74]
Hydrothermal treatment	BMP-2-CDs	BMP-2	7-10nm	Enhanced MG-63 cell biology and osteoinductive effects	[75]
Hydrothermal treatment	2-CDs	2-citric acid, poly (ethylene glycol) monomethyl ether (MW 550 Da), N, N-dimethylethylenediamine	16.8-18.6nm	Promotes cell proliferation (transforming growth factor-β) and cartilage matrix deposition (glycosaminoglycan, type II collagen); Inhibits undesirable type I and type X collagen deposition	[76]

In the above table”-” indicates that it is not mentioned in the relevant article

### Top-down approach

The top-down strategy involves using arc discharge, laser ablation, chemical oxidation, and ultrasonic synthesis to create CDs from macroscopic carbon structures such as graphite, activated carbon, and carbon nanotubes.

A top-down approach is used by relatively few CDs in bone defect repair applications, and we speculate that this is because bone defect repair frequently requires the addition of some ions and related cell growth-promoting components from a microscopic perspective, and the top-down approach is often not well suited to this requirement.

### Bottom-up approach

Microwave synthesis, thermal breakdown, hydrothermal treatment, template-based techniques, and plasma treatment are all used in bottom-up ways to make CDs from molecular precursors such citric acid, sucrose, and glucose. These approaches will be briefly described and their properties will be described in full below.

According to existing study data statistics, CDs used in bone defect healing are often produced from the bottom up. Among them, studies using the pyrolysis method account for the majority, and we speculate that it has now become the most widely used method for the synthesis of CDs, owing to advantages such as low cost, non-toxicity, environmental friendliness, and the ease with which the particles required for the study can be added to it [24, 77–79]. Microwave-assisted synthesis is the second most frequent synthesis technique. Microwave synthesis typically needs just carbohydrates, followed by the addition of inorganic ions, and the whole reaction step may be accomplished in a few minutes without the requirement of surface passivators, making it a popular method for CD synthesis [80–83].

## Application of carbon dots in the repair of bone defects

Carbon dots and modified carbon dots have been shown and extensively utilized in the area of biomedicine. CDs are becoming more important in biomedicine due to their distinctive hydroxyl and carboxyl structures on the surface, particularly in the healing of bone deformities, where CDs have piqued the interest of all researchers. As a result, we believe it is vital to highlight CDs' involvement in bone defect healing. We'll talk about them in words like anti-infection, osteogenic differentiation stimulation, and cell adhesion promotion.

### Scavenging free radicals

Because of their hydroxyl structure, CDs may rapidly scavenge harmful free radicals that cause oxidative stress damage and cell senescence, making them valuable for cell protection.

Using phloroglucinol and phenol as raw materials, Lu and colleagues used a one-step hydrothermal technique to manufacture biocompatible carbon dots (CDs-OH) with numerous hydroxyl groups [69]. The generated CDs-OH exhibits strong fluorescence, high photostability, and low cytotoxicity. According to LU et al, CDs-OH may also scavenge free radicals. As a free radical scavenger, it may significantly reduce the quantity of ROS in cell culture while also promoting cell growth and survival.

Das et al used a simple hydrothermal process to create carbon quantum dots (CQDs) doped SPIONS (FECD) [63]. According to their findings, the nanoparticles created have great cytocompatibility and blood compatibility. Wharton gel was used to grow bone marrow mesenchymal stem cells (MSCs), which were subsequently subcutaneously implanted. The generated tissue has osteogenic and chondrogenic stem cell differentiation capabilities in vivo. The MTT test was used to assess the short and long term cytocompatibility of FECD powder. The FECD sample's cell survival rate was found to be higher than the control sample's (tissue culture plate). The survival rate of FECD samples was up to 10 days, with the exception of the first day, which had significant advantages over the control group.

We hypothesized that the considerable cell survival in the experimental group with the addition of CDs was due to CDs' capacity to scavenge free radicals, and hence the addition of CDs might effectively restrict infection at the location of bone abnormalities and successfully promote cell survival.

### Promote cell adhesion and proliferation

Carbon dots' spherical form boosts the surface area to volume ratio, resulting in a larger contact area with cells, improved interaction between cells and more nutrients, and increased cell adhesion and proliferation.

Lu et al. prepared a novel cadmium-doped chitosan/nanohydroxyapatite (CS/nHA/Cd) scaffold by lyophilization method, which enhanced the adhesion, proliferation and osteogenic activity of rat bone marrow mesenchymal stem cells in vitro by upregulating local adhesion and osteogenic-related genes, and significantly promoted osteogenic differentiation and the formation of vascularized neointima at the fourth week (Fig. 3 [56],. As shown in Fig. 3, the expression of adherent spot kinase (FAK) and nucleoprotein (VCL) was significantly higher in CS/nHA/CD scaffolds than in CS/nHA scaffolds (p<0.01 and 0.05). the expression of PXN in CS/nHA/CD scaffolds was higher than in CS/nHA scaffolds, and the difference was statistically significant (p=0.054). These results suggest that cadmium-doped scaffolds promote the adhesion properties of rBMSCs through local adhesion and actin cytoskeleton pathways.

Transmembrane integrin receptors and intracellular proteins, including as FAK, PXN, and VCL, make up focal adhesion complexes [84]. FAK may influence alterations in actin and microtubule architecture, control cell-cell interactions, and recruit other focal contact proteins or their regulators [85]. PXL interacts with FAK and/or VCL and is linked to actin-based cytoskeletons, cell proliferation, and cell adhesion [86]. VCL interacts with actin, tensin, and PXL to play a role in cell adhesion, morphology, and growth [87]. These genes' up-regulation in focal signaling pathways causes the creation of focal adhesions, modifications to the actin cytoskeleton, and cell spreading. Additionally, rBMSCs attached in a flattened, spread-out form improve osteogenesis, according to a prior research [88].

**Fig. 4:**
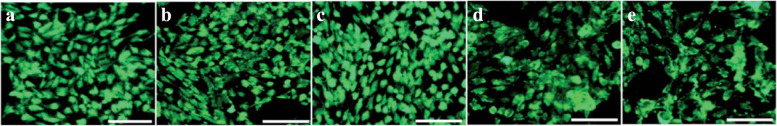
Fluorescent microscopic images showing MG 63 cells growing in tissue culture wells after 7 days of culture: (**a**) control (without nanomaterial), (**b**) HAP (100 μg mL^-1^), (**c**) CD (100 μg mL^-1^), (**d**) CD@HAP composite (100 μg mL^-1^) and (**e**) CD@HAP composite (200 mg mL^-1^). Scale bar represents 100 mm

Carbon dots modified hydroxyapatite (HAP) nanohybrids (Cd@HAP) were synthesized by hydrothermal method by Gogoi et al. Biological evaluation showed that the experimental group with added CDs showed significantly better cytocompatibility, cell proliferation and alkaline phosphatase activity on MG 63 osteoblasts than the other groups (Fig. 4 [54],.

**Fig. 5:**
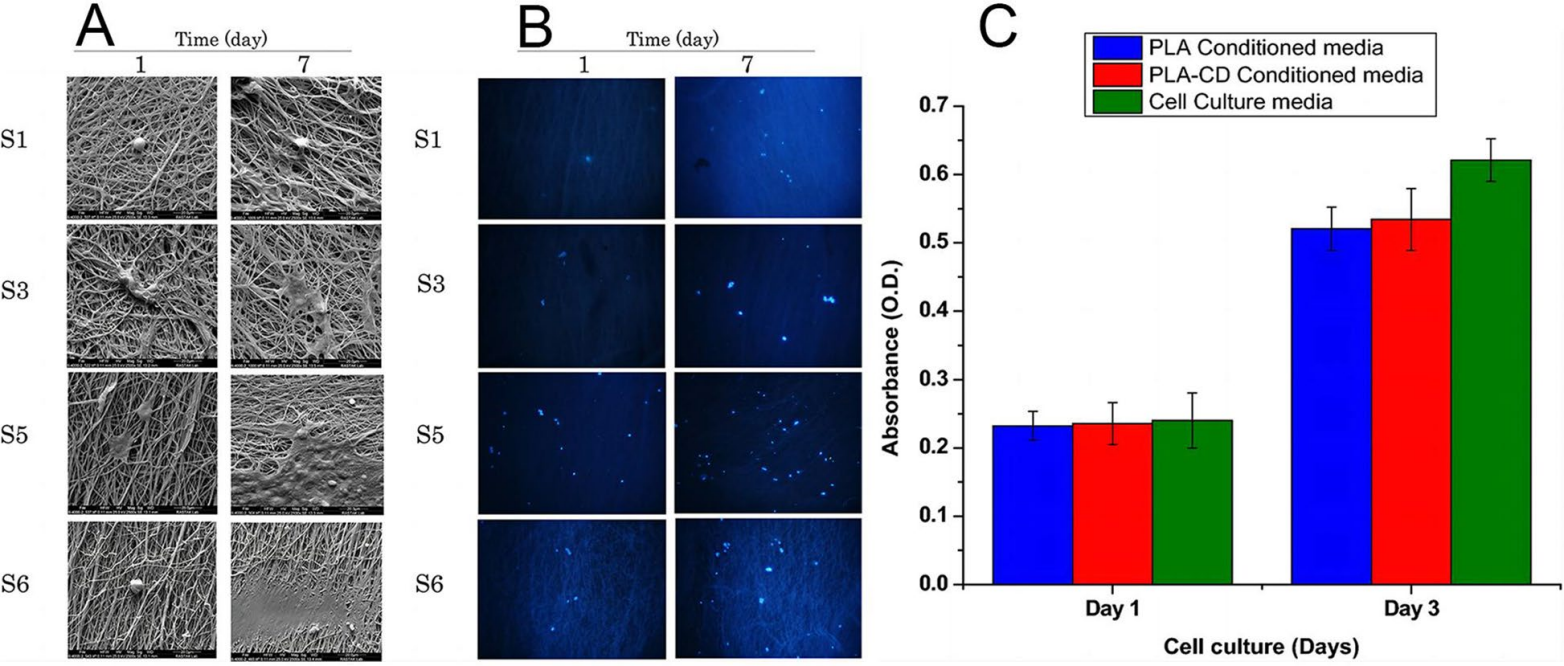
Cell changes on various carbon dots materials. **A**: Scanning electron microscope photos of MG-63 cells attached to the scaffold for 1 day and 7 days; **B**: DAPI staining fluorescence images of MG-63 cells cultured on scaffolds for 1 day and 7 days [66](© 2020 John Wiley & Sons Ltd); **C**: Study on cytotoxicity of polylactic acid and polylactic acid-cyclodextrin polymer [71]. (Copyright © 2021, The Author(s), under exclusive licence to Islamic Azad University)

Ghorghi et al created captopril (CP) supported polycaprolactone (PCL) / carbon quantum dots (CQDs) nanocomposite scaffolds for bone tissue regeneration using electrospinning and other methods [66]. Figure 5A displays the cell morphology during a seven-day period. On the first day of culture, the cells were attached to the scaffold and spherical in shape. After 7 days, the cells joined to form a larger matrix, and cell growth and adhesion rates were at their highest. The presence of DAPI nuclear staining in Fig. 5B indicates that MG-63 cells may survive and attach effectively to the scaffold. According to the results, CQDs increased cell survival and proliferation.

Using a hydrothermal / coprecipitation approach, Khajuria et al investigated the effect of nitrogen-doped carbon dots (NCDs) combined with hydroxyapatite (HA) nanoparticles NCDs-HA on the function of MC3T3-E1 osteoblasts and the osteogenic capability of the zebrafish (ZF) jaw regeneration (JBR) model. According to the results, NCDs-HA may induce osteogenesis via altering osteoblast proliferation, differentiation, and mineralization [58].

Dave et colleagues created a biodegradable polymer nanocomposite using polylactic acid and CDs [71]. The biocompatibility of PLA and PLA-CD scaffolds was evaluated using cell growth. Because the fusion layer of Saos-2 cells was visible on PLA-CD scaffolds, researchers determined that they had better cell adherence than PLA scaffolds. This observation is consistent with the findings of the MTS cell proliferation assay. Figure 5C reveals that polylactic acid-CD scaffolds outperformed polylactic acid scaffolds in terms of cell proliferation.

### Regulatory genes induce mineralization and vascularization

Jin et al created ascorbic acid carbon dots in a single microwave process (CDs) [64]. The results indicated that CDs may effectively stimulate matrix mineralization, osteogenic differentiation, and new bone regeneration in a vivo skull lesion model in vitro.

**Fig. 6:**
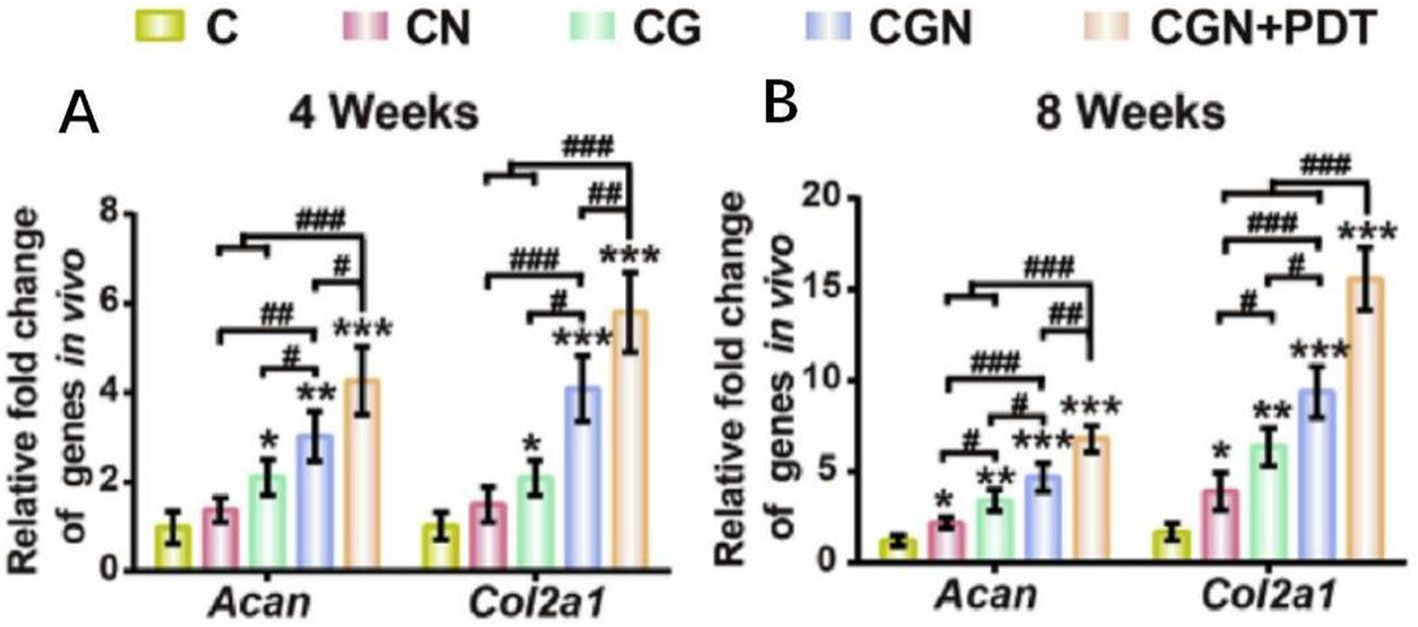
(**A**, **B**) expression of chondrocyte-specific genes Acan and Col2a1 at 4 and 8 weeks after subcutaneous implantation in nude mice [57] (C = collagen, CN = collagen mixed with CD NPs, CG = collagen crosslinked with genipin, CGN =collagen crosslinked with genipin and CD NPs, CGN + PDT=CGN after 808 nm laser irradiation at a power density of 8.3 mW/cm2 for 3 min). (© 2019 Elsevier Ltd. All rights reserved)

Lu et al. combined biocompatible carbon dots nanoparticles (CDNPs) with collagen to form an injectable hydrogel (called collagen-genipin-CD nanoparticles, CGN) using a natural product crosslinker (genipin) [57]. The expression of cartilage-specific genes in newborn tissues was determined using qRT-PCR. Figure 6 shows that the cartilage-specific expression levels of Acan2 and Col2a1 in the CGN group were higher than those in the collagen group at each time point. Concurrently, the mineralization of bone injury was seen. According to the results, cadmium sulfide may increase bone mineralization and regeneration by altering the expression of certain genes.

As raw materials, Gogoi et al employed carbon dots and four different peptides (tannic acid, tannic acid, tannic acid, and tannic acid) to create water dispersible hyperbranched polyurethane (SVVYGLR,PRGDSGYRGDS) [53]. The study revealed that biological nano-hybridization of carbon dots and a collection of peptides may enhance osteoblast proliferation and differentiation, induce angiogenesis, and improve bone conductivity and bone differentiation capabilities of the materials. MG63 cell adhesion, proliferation, and differentiation on biological nanocomposites were all enhanced, demonstrating the biological nano-significance hybrid's role in bone repair. Mineralization and vascularization studies, on the other hand, have indicated that calcification and angiogenesis improve bone recovery.

### Antibacterial properties

Bacterial infection is the primary problem in the repair of infective bone abnormalities. Many studies are currently being carried out to coat the implant surface with antibiotics in order to prevent the formation of infectious biofilm and enhance bone repair. Toxic drugs, on the other hand, not only promote the development of multidrug-resistant bacteria but also reduce the osteogenic activity of scaffold materials. As a consequence, it is necessary to find a novel and efficient method of dealing with infection during the bone defect operation. Carbon dots have been widely used in biomedicine since research indicated that they had powerful anti-oxidation and anti-inflammatory capabilities.

**Fig. 7:**
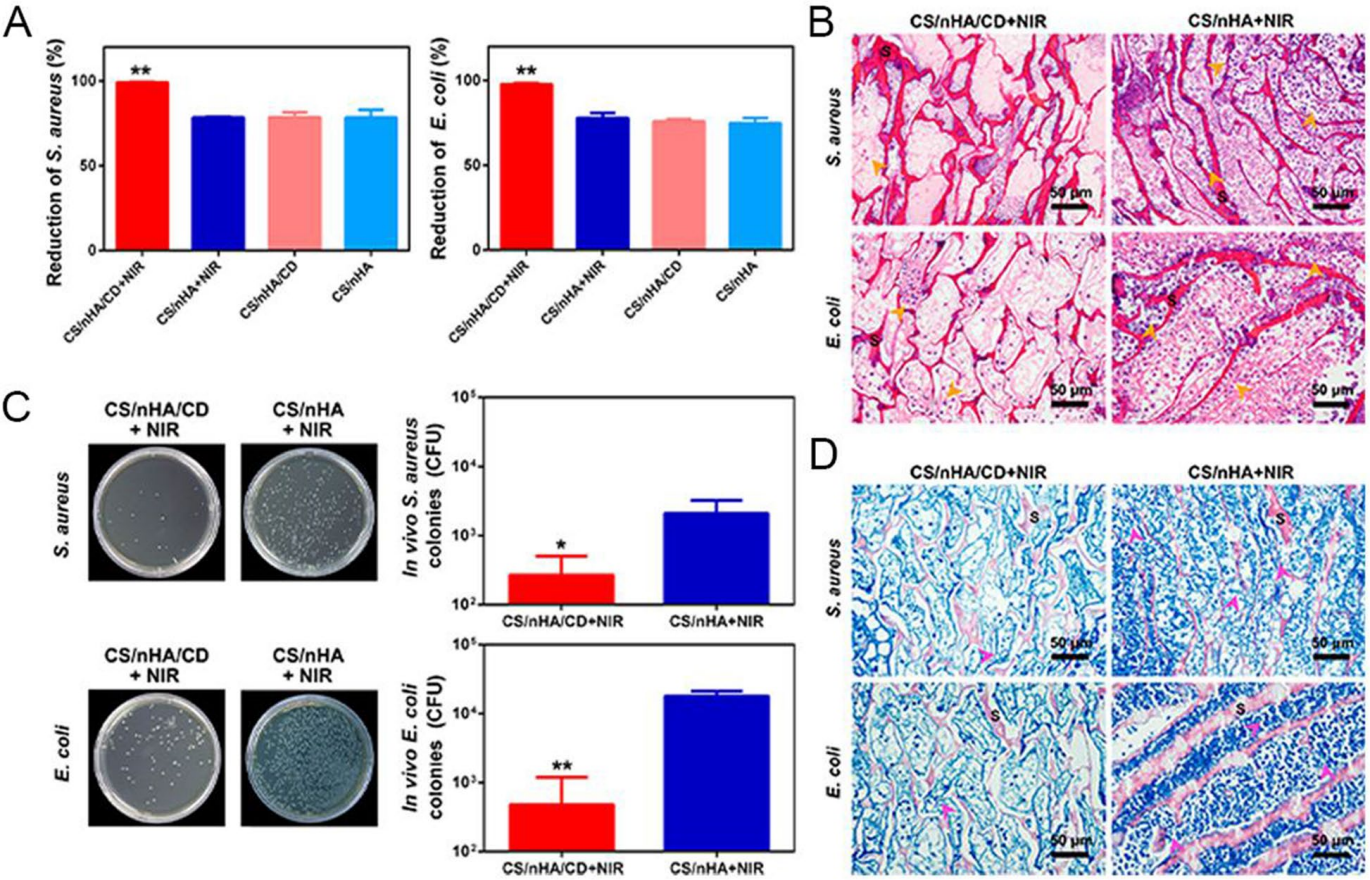
Antimicrobial performance of the stents. (**A**) The effectiveness of several groups' in vitro antibacterial treatments against clinically significant Staphylococcus aureus (left) and Escherichia coli (right). The antibacterial activity against clinical microorganisms was greater in the CS/NHA/CD+NIR group. The mean and standard deviation of the values are shown; *p<0.05, **p<0.01. (**B**) H&E staining of samples collected from the various groups. The CS/nHA/CD+NIR group had a limited number of lobulated neutrophils (orange arrows), while the CS/nHA+NIR group had a large number of lobulated neutrophils. (**C**) After the specimens were taken, they were given in vivo treatment for a week, and then they were incubated for 24 hours. The number of colonies of clinically relevant S. aureus (top) and E. coli (bottom) were then counted. The mean and standard deviation of the values are shown; *p<0.05, **p<0.01. (**D**) Samples stained with Giemsa. Less bacteria in the CS/NHA/CD+NIR group (pink arrows), more bacteria in the CS/NHA+NIR group (blue arrows). Scaffold is represented by S [56]

Unlike carbon dots, which offer anti-infection and free radical scavenging properties. Carbon dots that have been modified with various functional groups may subsequently be used to perform an efficient anti-infection role in bone defect healing. Lu et al. used clinically obtained Staphylococcus aureus and Escherichia coli scaffolds to test the impact of NIR on the antibacterial efficacy of CDs by adjusting the presence or absence of near-infrared light (NIR) (Fig. 7A) [56]. The additional CDs were shown to have clear antibacterial capabilities, with inhibition rates of 99 percent and 97 percent against the harmful bacteria S. aureus and E. coli, respectively, whereas the antibacterial rates of the other groups were about 75 percent. More notably, the scaffold's antibacterial activity under NIR light boosted its antibacterial action. When the in vivo antimicrobial effect of photothermal therapy (PTT) was investigated later, as shown in Fig. 7C, Groups that use only NIR still had a large number of S. aureus and E. coli colonies, whereas the group where NIRs and CDs are applied simultaneously had a significant reduction in the number of bacteria, indicating that the addition of CDs could significantly inhibit bacterial growth.

H&E examination of the specimens (Fig. 7B) revealed that the group without CDs had a high number of lobulated neutrophils (orange arrows) owing to inflammation and injected bacteria. The group with additional CDs, on the other hand, exhibited very little inflammation, which was owing to the improved antibacterial action after NIR irradiation. Furthermore, Giemsa staining revealed the existence of bacteria in the group without CDs (Fig. 7D), but no visible bacteria were discovered in the group with CDs, showing that the addition of heat favored the suppression of bacterial growth. Thus, the scaffolds containing CDs following NIR radiation had the strongest antibacterial activity, indicating that light conditions and CDs may effectively cooperate to remove clinically important bacterial infections.

Geng et al. proposed for the first time that altering the surface charge of carbon quantum dots (CQDs) may be used to control their antibacterial and osteogenic properties. Positively charged CQDs (p-CQDs) show excellent antibacterial activity and the ability to inhibit biofilm formation, according to Geng et al's study [72].

**Fig. 8:**
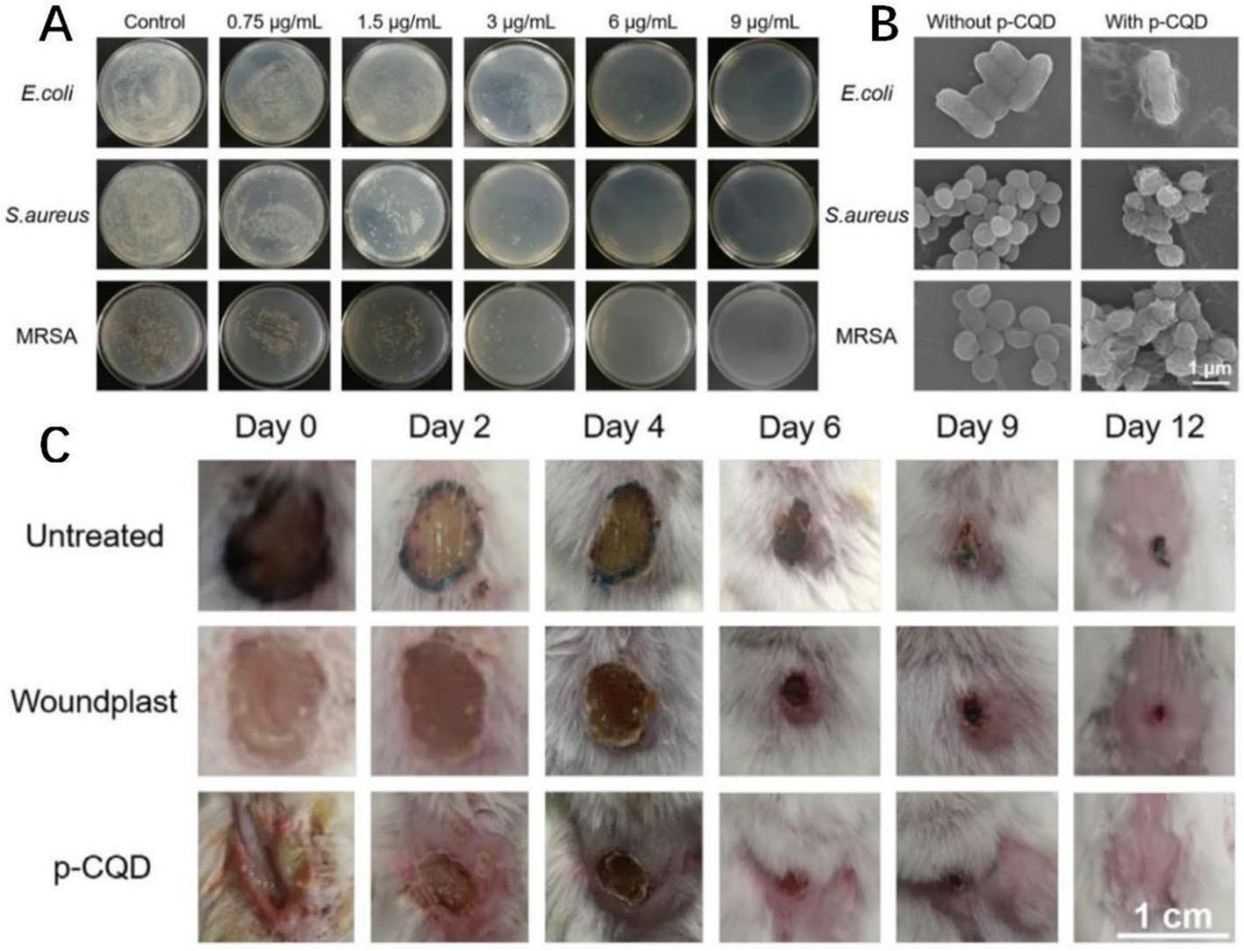
Antibacterial effect of cationic carbon dots. (**A**) Escherichia coli, Staphylococcus aureus and MRSA treated with different concentrations of p-CQD (0e9mg/mL) showed typical colony formation on LB Agar plate. (**B**) scanning electron microscope images of Escherichia coli, Staphylococcus aureus and methicillin-resistant Staphylococcus aureus before and after p-CQDs treatment.(**C**): typical skin wound photos of MRSA-infected mice treated with PBS solution (control), commercial wound plastic or p-CQDs for 0, 2, 4, 6, 9 and 12 days [72].( © 2021 Elsevier Ltd. All rights reserved.)

As shown in Fig. 8A, increasing the concentration of p-CQDS from 0 mg/mL to 9 mg/mL results in a substantial inhibitory impact against all three species of bacteria. On an LB Agar plate for gram-negative bacteria, no colony was identified at a concentration of 6 mg/mL of p-CQDs (Escherichia coli). Figure 8B depicts scanning electron microscope images of Escherichia coli, Staphylococcus aureus, and methicillin-resistant Staphylococcus aureus. The number of germs in the p-CQD group was much lower than in the control group, indicating that p-CQD exhibited excellent antibacterial potential. Figure 8C demonstrates rat wound infection with different materials. The frequency of wound infection is substantially lower in the p-CQDs group than in the other groups, indicating that p-CQDs has a definite antibacterial effect [72].

The antibacterial activity of p-CQDs was also investigated. The prevalence of Staphylococcus aureus (S. aureus) and methicillin-resistant Staphylococcus aureus (MRSA) was significantly lower in the P-CQD group than in the control group. According to the results, positively charged p-CQDs may kill multidrug-resistant bacteria while also reducing biofilm formation.

The above study illustrates that the addition of CDs can significantly increase the antimicrobial effect of the scaffold material, and this antimicrobial effect can be further enhanced with the presence of light conditions.

### Increase alkaline phosphatase activity

The glycoprotein alkaline phosphatase (ALP) is located on the surface of cells. Increased ALP levels in bone defects are an early phenotypic indicator of osteogenic differentiation, indicating that bone regeneration is taking place [55, 70, 89]. Previous study has shown that carbon dots and their composite products significantly increase the level of ALP in bone defects, and bone regeneration has been active in the follow-up experiment.

Geng et al created biocompatible and positively charged near infrared sensitive carbon dots using an ultrafast microwave-assisted hydrothermal technique (CDs) [68], Geng et al reported that hMSCs treated with Cd/WS2 had considerably greater alkaline phosphatase (ALP) activity than the control group, demonstrating that the presence of CDs might enhance osteogenic differentiation and bone regeneration.

**Fig. 9:**
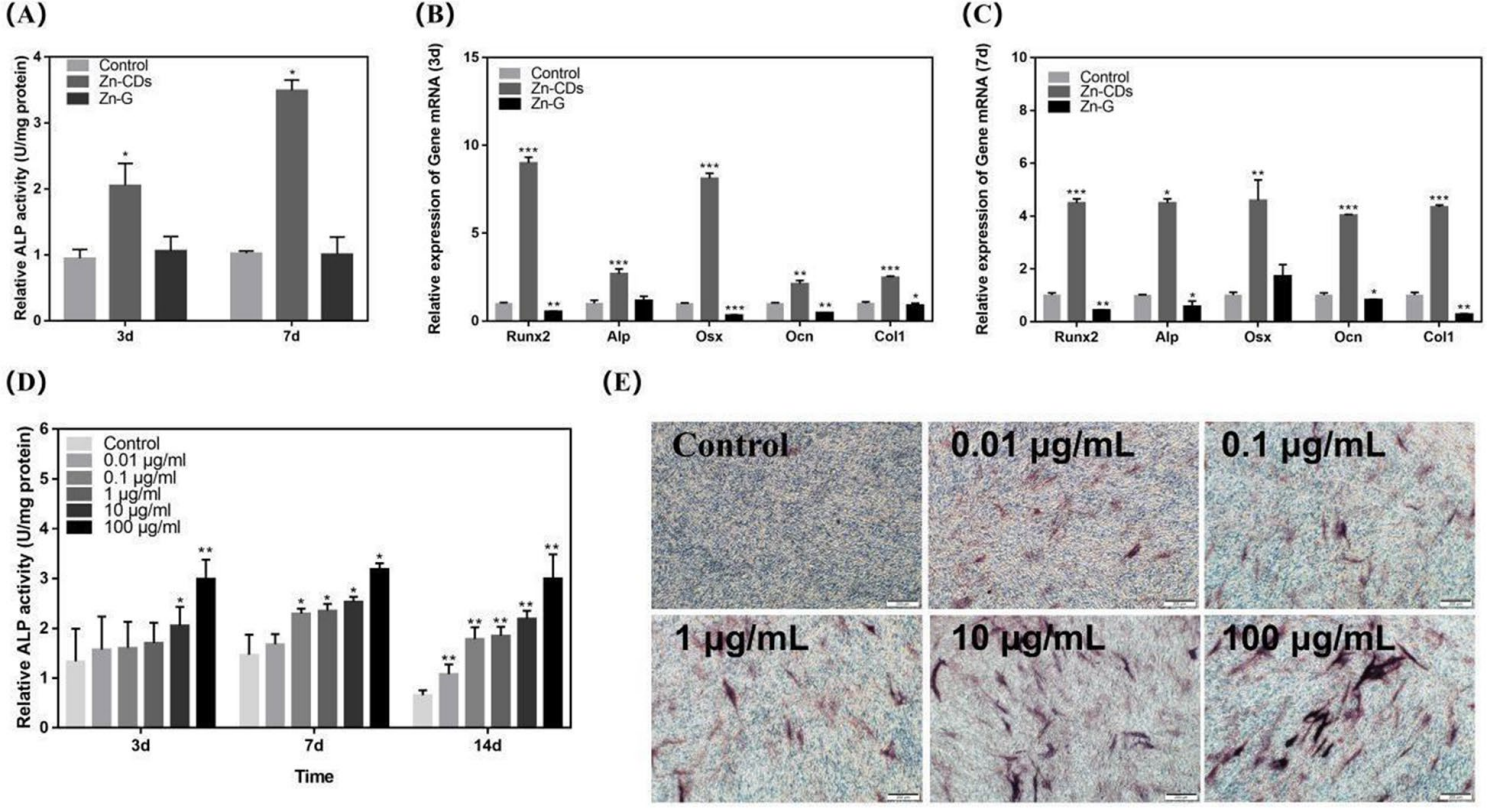
Different groups' effects on alkaline phosphatase activity. (**A**) the relative alkaline phosphatase activities of control group, Zn-CDs group and Zn-G group were compared on the 3rd and 7th day (concentration was 100 μg/mL); (**B**) Compare the expression of five related genes in MC3T3-E1 cells after incubation of Zn-CDs and Zn-G (100μg/mL) for 3d and (**C**) 7d; (**D**) ALP activity. The effect of Zn-CDs on ALP activity was dose-dependent. (*)*P*<0.001, (**)*P*<0.01, *P*<0.001; (**E**) ALP staining results showed that Zn-CDs had an effect on alkaline phosphatase activity of MC3T3-E1 cells on the 14th day [60]

Meng and colleagues developed a unique kind of bifunctional Zn2+ doped carbon dots using a one-step hydrothermal method (Zn-Cd) [60]. Figure 9 shows that the ALP expression of Zn-Cd was 4.5 times higher than that of the control group on the seventh day. This reveals that Zn-Cd may significantly boost ALP activity. Meng et al observed that Zn-Cd has a significant capacity to promote osteoblast differentiation, implying that it has a greater potential to induce osteogenesis than the control group and may aid in bone regeneration.

### Promotes osteogenic differentiation

In biomedicine, the investigation of carbon point processes has long been popular. The present research found that the presence of CDs greatly accelerated the process of osteogenic differentiation by upregulating bone matrix mineralization-related gene expression and osteoblast development. Additionally, CDs may boost intracellular calcium ions and activate the PERK-eIF2-ATF4 and endoplasmic reticulum stress pathways, which in turn can promote osteoblast production and bone repair. We now have a microscopic understanding of how CDs encourage osteogenic differentiation and subsequently bone defect repair.

**Fig. 10:**
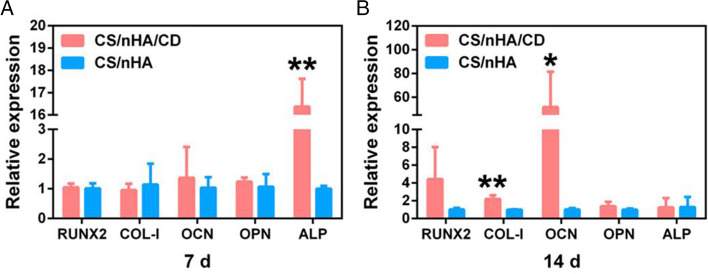
(**A**, **B**) Gene expression levels associated with osteogenesis after 7 and 14 days in culture. On day 7, ALP expression levels in the CS/NHA/CD scaffold were considerably greater than those in the CS/NHA scaffold, and on day 14, Col-1 and OCN expression levels in the CS/NHA/CD scaffold were significantly higher than those in the CS/NHA scaffold [56]

As previously stated, Lu et al. revealed that the CS/nHA/CD scaffold may promote cell adhesion and proliferation via regulating related genes. Furthermore, Lu et al. observed that CS/nHA/CD scaffolds could significantly increase the mRNA expression levels of osteogenic-related genes in rBMSCs in the scaffolds, as well as alkaline phosphatase (ALP) gene expression levels (Fig. 10 [56],. Early alkaline phosphatase activity is an important sign of osteogenic differentiation, implying that rBMSCs in the CS/nHA/CD scaffold grow into osteoblasts sooner than rBMSCs in the CS/nHA scaffold alone. As the study progressed, type I collagen (Col-I) and osteocalcin (OCN) expression levels in the CS/nHA/CD scaffold alone were considerably higher than in the CS/nHA scaffold alone. This shows that the addition of CDs stimulated bone matrix synthesis by increasing the expression of key genes involved in osteoblast growth and bone matrix mineralization. This study shows how CDs may stimulate osteogenic differentiation and, as a result, bone defect repair.

The findings show that CDs can promote osteogenic differentiation by regulating the mRNA expression levels of osteogenic-related genes and increasing the expression levels of alkaline phosphatase (ALP) genes, which promotes osteogenic differentiation, indicating that CDs have a significant advantage in promoting osteogenic differentiation.

Although it has been demonstrated that modified CDs can often play a positive role in bone defect repair and effectively upregulate the expression of related genes to promote osteogenic differentiation, the mechanism of CDs themselves in promoting osteogenic differentiation has yet to be fully investigated, and we have yet to link their effects to the CDs' surface motifs, nor have we been able to systematically elucidate this. We have not yet been able to link their effects to the surface groups of CDs, nor have we been able to interpret them systematically.

However, biomaterials such as metal nanoparticles, particularly magnesium ions and zinc ions, have been extensively employed in the therapy of bone abnormalities, and their methods of action have progressively been elucidated. As a result, we want to offer the study in Table 2 to demonstrate their effects and processes in a more accessible manner, and then make a meaningful comparison with the CDs and their modified CDs in this publication, pointing out the route for future CD research in bone defect healing.

**Table 2:** The contribution of metal ions to bone and their mode of action

	Role	Mechanism of action	References
Li^+^	Osteogenesis	Lithium has the ability to block the expression of GSK3, a blocker of the Wnt signaling pathway. Other studies showed that lithium acts as an agonist of the Wnt/b-catenin pathway to enhance fracture healing.	[90]
Zn^2+^	Osteogenesis	It has been discovered that zinc participates in the structural, catalytic, or regulatory aspects of ALP expression, where it is crucial for osteogenesis and mineralization. Additionally, it is thought that zinc has the power to stop the osteoclastic resorption process.	[91–94]
Mg^2+^	Osteogenesis, Angiogenesis, Neural stimulation	Magnesium stimulates the synthesis of HIF and PGC-1α in undifferentiated and differentiated hBMSCs, respectively. This promotes the VEGF synthesis. Mg^2+^ penetrates DRG neurons, induces the release of CGPR, and subsequently activates PDSCs to produce the osteogenic differentiation-related genes.	[95–99]
Sr^2+^	Osteogenesis	Strontium stimulates the activity of osteoblastic cells while decreasing the activity of osteoclasts. It stimulates CaSR and signaling pathways downstream. It enhances the synthesis of OPG and reduces the expression of RANKL. This stimulates osteoblast proliferation, differentiation, and viability and causes osteoclast death, which decreases bone resorption.	[100, 101]
Cu^+^	Angiogenesis, Osteogenesis	Copper is known to be a factor that induces angiogenesis by imitating hypoxia. Cu2-induced immunological milieu may indirectly promote robust osteogenic differentiation of BMSCs by activating the Oncostation M (OSM) pathway.	[102, 103]
Co^2+^	Angiogenesis	Co^2+^ ion is hypothesized to stimulate hypoxia cascade formation, hence stabilizing HIF-1α. The cells will then produce genes (such as VEGF and EPO) that promote neovascularization and angiogenesis in response to this hypoxic environment.	[104–106]
Si^4+^	Angiogenesis, Osteogenesis	Silicon has been demonstrated to stimulate angiogenesis by upregulating NOS, resulting in an increase in VEGF production at low concentrations when human dermal fibroblasts are cultivated. Osteogenic process is not fully known. However, it has been shown that Si4 at greater concentrations plays a crucial part in the mineralization process.	[107, 108]

Reprinted from Ref. [33]

## Conclusion and prospect

Infection in the defect location, poor biocompatibility of the filling material, and delayed osteogenic differentiation of local bone tissue all impede the healing of bone defects. CDs are a unique nanomaterial that has the potential to be used in bone regeneration, spinal cord injury repair, and wound healing [91–95]. As previously stated, carbon dots and modified carbon dots have anti-infection, antibacterial, gene regulation for osteogenic differentiation, and enhanced alkaline phosphatase activity, which may greatly improve bone defect healing. In bone defect repair, the use of CDs may successfully solve the issues of infection, poor biocompatibility of filler materials, and delayed osteogenic differentiation of local bone tissue. However, existing studies on the microstructure of carbon dots are not very comprehensive; for example, we still cannot accurately link the hydroxyl and carboxyl groups on the surface of CDs with genes and proteins such as those regulating osteogenic differentiation, which will be the main research direction in this field in the future. We hope that further study on CDs will give more precise assistance for bone defect healing in the future.

## Data Availability

Not applicable.
